# Serum Progesterone Elevation Adversely Affects Cumulative Live Birth Rate in Different Ovarian Responders during *In Vitro* Fertilization and Embryo Transfer: A Large Retrospective Study

**DOI:** 10.1371/journal.pone.0100011

**Published:** 2014-06-13

**Authors:** Zhiqin Bu, Feifei Zhao, Keyan Wang, Yihong Guo, Yingchun Su, Jun Zhai, Yingpu Sun

**Affiliations:** 1 Reproductive Medical Center, First Affiliated Hospital of Zhengzhou University, People's Republic of China; 2 School of Public Health, Zhengzhou University, People's Republic of China; Institute of Zoology, Chinese Academy of Sciences, China

## Abstract

In order to explore the relationship between serum progesterone (P) level on the day of human chorionic gonadotrophin (HCG) administration and cumulative live birth rate in patients with different ovarian response during *in vitro* fertilization (IVF), we carried out this retrospective cohort study including a total of 4,651 patients undergoing their first IVF cycles from January 2011 to December 2012. All patients with a final live birth outcome (4,332 patients) were divided into three groups according to ovarian response: poor ovarian responder (≤5 oocytes, 785 patients), intermediate ovarian responder (6–19 oocytes, 3065 patients) and high ovarian responder (≥20 oocytes, 482 patients). The thresholds for serum P elevation were 1.60 ng/ml, 2.24 ng/ml, and 2.50 ng/ml for poor, intermediate, and high ovarian responders, respectively. Cumulative live birth rate per oocyte retrieval cycle was calculated in each group. The relationship between serum P level and cumulative live birth rate was evaluated by both univariate and multivariate logistic regression analysis. Cumulative live birth rate per oocyte retrieval cycle was inversely associated with serum P level in patients with different ovarian response. For all responders, patients with elevated P level had significantly higher number of oocytes retrieved, but lower high quality embryo rate, and lower cumulative live birth rate compared with patients with normal serum P level. In addition, serum P level adversely affected cumulative live birth rate by both univariate and multivariate logistic regression analysis, independent of ovarian response. Serum P elevation on the day of HCG administration adversely affects cumulative live birth rate per oocyte retrieval cycle in patients with different ovarian response.

## Introduction

The relationship between serum progesterone (P) level on the day of human chorionic gonadotropin (HCG) administration and outcome of *in vitro* fertilization (IVF)/intracytoplasmic sperm injection (ICSI) and embryo transfer (ET) has been controversial for several decades [1], [2], [3], [4], [5], [6]. Most studies have evaluated the association between serum P level and clinical outcome in fresh IVF/ICSI cycles, and advocated that serum P elevation on the day of HCG administration may adversely affect clinical outcome by jeopardizing endometrial receptivity [7], [8], [9]. However, embryo cryopreservation is now in common use all over the world, giving clinicians the opportunity to use surplus embryos in frozen-thawed embryo transfer (FET) cycles. Thus, many fertility centers suggest their patients with serum P elevation to wait for embryo transfer in next FET cycles. Since the frozen embryos are also produced in fresh cycles, in order to evaluate the impact of serum P elevation on outcome of an IVF/ICSI cycle, it would be more logical to consider the cumulative live birth rate from the fresh and all FET cycles combined, instead of merely looking at the single fresh cycle outcome.

Another question to address is the relationship between serum P level and ovarian response. Recent studies have shown that serum P level is positively associated with ovarian response [10], [11]. Thus, it is reasonable and important to assess the relationship between serum P level and IVF/ICSI outcome according to different ovarian response.

Therefore, the purpose of the present study is to investigate the relationship between serum P level on the day of HCG administration during IVF/ICSI and the cumulative live birth rate per oocyte retrieval cycle in patients with different ovarian response.

## Materials and Methods

### Patients

This retrospective cohort study included 4,651 patients undergoing their first IVF/ICSI cycles carried out between January 2011 and December 2012 at the Reproductive Medicine Center, First Affiliated Hospital of Zhengzhou University, China. Cycles carried out for pre-implantation genetic diagnosis (PGD) or those with donor gametes were excluded from this analysis. All patients signed written informed consent. Institutional Review Board of First Affiliated Hospital of Zhengzhou University approved this study.

### Controlled ovarian hyperstimulation protocols

Pituitary down-regulation was performed with a standard long GnRH agonist protocol, (modified) super long GnRH agonist protocol, or GnRH antagonist protocol, as shown in previous studies [12], [13]. The selection of the protocol and the dose of gonadotrophin were individualized according to each patient's basic information and clinician's preference.

Embryo quality evaluation was done on the cleavage stage. The grading criteria were described somewhere else [14]. Grade 1 and grade 2 embryos were considered to be high quality embryos. Embryo transfer took place between 2 and 6 days after oocyte retrieval. The number of embryos transferred complied with national regulations and patient's ovarian response and requests. A maximum of three embryos can be transferred.

Cryopreservation was performed 3–6 days after oocyte retrieval. The details of the freezing and thawing protocols in our center were reported previously [15].

The definitions of some clinical parameters in this study are shown as follows: Fertilization rate  =  number of 2 PN (pronuclear)/number of oocytes retrieved. Available embryo rate  =  number of available embryos/number of oocytes retrieved. High quality rate  =  number of high quality embryos/number of oocytes retrieved.

### Data Collection

Data were obtained from computerized databases. Patient characteristics were evaluated, including age, body mass index (BMI), infertility diagnosis, infertility duration, and basal serum follicular stimulation hormone (FSH) level. Other parameters obtained from each cycle were also recorded, including total duration and dose of gonadotropins, peak serum estradiol level, luteinizing hormone (LH) level, and P level on the day of HCG administration; oocytes obtained per cycle, fertilization rate, number of available embryos, number of high quality embryos, and live births. The primary outcome was the cumulative live birth in the fresh and all FET cycles combined following the same index stimulation cycle.

### Statistical analysis

Patients were categorized into three groups based on ovarian response: poor ovarian responder (≤5 oocytes obtained), intermediate ovarian responder (6–19 oocytes obtained), and high ovarian responder (≥20 oocytes obtained). In all the three ovarian response groups, patients were then divided into six distinct groups based on serum P level on the day of HCG administration: ≤0.49, 0.50–0.99, 1.00–1.49, 1.50–1.99, 2.00–2.49, and ≥2.50 ng/mL. Serum P elevation on the day of HCG administration was defined as cumulative 95% of P level in each ovarian response group: poor ovarian responder (≥1.60 ng/mL), intermediate ovarian responder (≥2.24 ng/mL), and high ovarian responder (≥2.50 ng/mL).

Cumulative live birth rate was calculated for each P interval. The spearman order correlation coefficients were used to determine the relationship between serum P level and patient basic characteristics. Demographic and clinical characteristics in groups with and without P elevation were compared using Student T test and Chi-square test, as appropriate. Univariate logistic regression analysis was used to explore the association between all factors and cumulative live birth. And those having significant association with cumulative live birth by univariate analysis were included into multivariate logistic regression for further analysis.

Statistical analysis was carried out using the Statistical Program for Social Sciences (SPSS Inc., Version 17.0, Chicago, IL, USA). In all cases, the value of *P*<0.05 was considered statistically significant.

## Results

### Patient Characteristics

The patients' basic information is summarized in Table 1. In the 4,651 patients undergoing their first IVF/ICSI cycles, 4,332 patients had a final cumulative live birth outcome (2,518 patients reached a live birth, and 1,814 patients did not and had no embryos left). The overall cumulative live birth rate in these 4,332 patients was 58.18%.

**Table 1:** Basic characteristics of the 4,651 patients undergoing their first IVF/ICSI cycle.

Parameter	n = 4651
Age (y)	30.82±4.78
BMI (kg/m^2^)	22.25±3.06
Duration of infertility (y)	4.75±3.72
Basal FSH (IU/L)	7.76±2.74
Infertility diagnosis	
Tubal factor	1149 (24.7%)
Ovulation disorder	818 (17.6%)
Endometriosis, pelvic and uterine factors	1052 (22.6%)
Diminished ovarian reserve	172 (3.7%)
Male factor	1223 (26.3%)
Unexplained and other	237 (5.1%)
Protocols	
Standard long protocol	3923 (84.3%)
(Modified)Super-long protocol	278 (6.0%)
GnRH-antagonist/short/protocol	450 (9.7%)
Live birth outcome	
Yes-≥1 live birth babies	2518
No-no embryo left	1814
No-≥1 embryos left	300
No-ongoing pregnancy	19

Note: values are the mean ± SD unless otherwise noted. BMI, body mass index; FSH, follicular stimulation hormone; GnRH, gonadotropin releasing hormone.

Table 2 shows the relationship between serum P level on day of HCG administration and patient's basic and clinical parameters. Basing on simple regression analysis of the pooled data, serum P level was most positively correlated with number of oocytes retrieved (r = 0.27, *P* = 0.00), serum E_2_ level on day of HCG administration (r = 0.41, *P* = 0.00).

**Table 2:** The relationship between serum P level on day of HCG administration and patient's basic and clinical characteristics in 4,651 patients.

Parameter	Pearson Correlation	*P* value
Age (y)	0.000	0.975
BMI (Kg/m^2^)	−0.071	0.000
Duration of infertility (y)	0.019	0.206
Basal FSH (IU/L)	−0.143	0.000
Gn duration (days)	0.003	0.822
Gn dosage (IU)	0.092	0.000
Peak E_2_ (pg/ml)	0.407	0.000
Peak LH (IU/L)	−0.041	0.006
No. of retrieved oocytes	0.269	0.000

Note: BMI, body mass index; FSH, follicular stimulation hormone; Gn, gonadotropin; E_2_, estrogen; LH, luteinizing hormone.

### Poor ovarian responders

**Figure 1:**
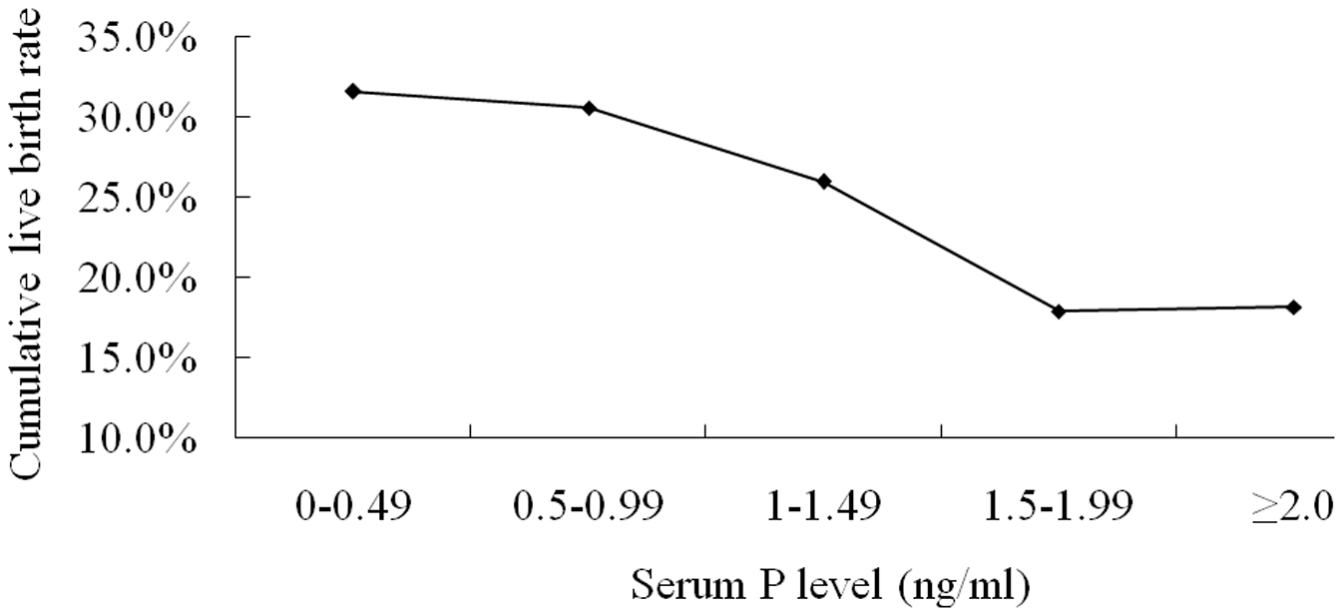
Relationship between serum P level on the day of HCG administration and cumulative live birth rate per oocyte retrieval cycle in poor ovarian responders (retrieved oocytes ≤5).

Figure 1 shows the association between cumulative live birth rate and serum P level in poor ovarian responders (n = 785). Cumulative live birth rate negatively associated with serum P level, and dropped dramatically when serum P≥1.50 ng/mL. Compared with patients in serum P elevation (P≥1.50 ng/mL) group, those with serum P non-elevation (P<1.50 ng/mL) had significantly higher FSH levels, and lower peak E_2_ levels. Meanwhile, number of oocytes retrieved was lower in non-elevation group, even though the difference did not reach statistical significant. However, the results show that number of high quality embryos, available embryo rate, high quality embryo rate, and cumulative live birth rate were higher in the non-elevation group than those in the serum P elevation group (Table 3).

**Table 3:** Patients' characteristics and cycle outcomes in poor ovarian responders (retrieved oocytes ≤5) with and without P elevation (≥1.6 ng/ml) on day of HCG administration.

	Non-elevated P (n = 747)	Elevated P (n = 38)	*P* value
Age (y)	33.8±5.2	31.6±5.0	0.011
BMI (Kg/m^2^)	22.3±3.0	21.5±2.5	NS
Duration of infertility (y)	5.8±4.2	5.6±4.1	NS
Basal FSH (IU/L)	9.7±4.3	8.3±2.4	0.002
Gn duration (days)	11.43±2.8	11.42±2.6	NS
Gn dosage (IU)	2868±1135	2917±1355	NS
Peak E_2_ (pg/ml)	2220±1306	3118±1588	0.000
Peak LH (IU/L)	2.6±2.3	4.0±7.6	NS
No. of retrieved oocytes	3.44±1.3	3.79±2.25	NS
Fertilization rate (%)	71.1±26.9	66.5±29.8	NS
No. of available embryos	2.1±1.1	1.8±1.1	NS
No. of high quality embryos	1.8±1.1	1.3±0.8	0.010
Available embryo rate (%)	64.3±27.9	50.4±26.9	0.003
High quality embryo rate (%)	55.0±31.3	38.4±27.2	0.001
Cumulative live birth rate (%)	227/747 (30.4)	7/38 (18.4)	NS

Note: values are the mean ± SD unless otherwise noted. NS, not significant.

BMI, body mass index; FSH, follicular stimulation hormone; Gn, gonadotropin; E_2_, estrogen; LH, luteinizing hormone.

Univariate logistic regression showed that patient age, duration of infertility, basal FSH, number of oocytes retrieved, fertilization rate, number of available embryos, number of high quality embryos, available embryo rate, high quality embryo rate, and serum P level on the day of HCG administration were significantly associated with cumulative live birth rate in poor ovarian responders. However, after controlling for related factors in multivariate logistic regression analysis, only patient age, duration of infertility, and serum P level were associated with cumulative live birth rate (Table 4).

**Table 4:** Factors associated with cumulative live birth rate in different ovarian responders by logistic regression.

	Poor ovarian responders		Intermediate ovarian responders		High ovarian responders	
	COR (95% CI)	AOR (95% CI)	COR (95% CI)	AOR (95% CI)	COR (95% CI)	AOR (95% CI)
Age (y)	0.88 (0.85–0.91)**	0.89 (0.85–0.93)**	0.92 (0.90–0.93)**	0.94 (0.92–0.96)**	0.98 (0.93–1.04)	
BMI (Kg/m^2^)	0.99 (0.94–1.05)		0.97 (0.95–1.00)		1.07 (0.99–1.15)	
Duration of infertility (y)	0.90 (0.86–0.94)**	0.92 (0.87–0.98)**	0.92 (0.90–0.94)**	0.96 (0.94–0.99)**	0.91 (0.84–0.97)**	0.91 (0.84–0.98)*
Basal FSH (IU/L)	0.96 (0.92–1.00)*		0.95 (0.92–0.98)**		1.05 (0.92–1.21)	
Gn duration (days)	0.97 (0.92–1.03)		0.95 (0.92–0.99)*		0.96 (0.85–1.10)	
Gn dosage (IU)	1.00 (0.99–1.00)		1.00 (0.99–1.00)		1.00 (0.99–1.00)	
Peak E_2_ (pg/ml)	1.00 (1.00–1.00)		1.00 (1.00–1.00)		1.00 (1.00–1.00)	
Peak LH (IU/L)	0.98 (0.92–1.04)		1.04 (0.98–1.11)		1.01 (0.74–1.38)	
No. of retrieved oocytes	1.45 (1.28–1.65)**		1.08 (1.06–1.11)**		1.02 (0.98–1.07)	
Fertilization rate (%)	2.14 (1.20–3.84)**		4.58 (3.06–6.85)**		21.1 (5.4–82.6)**	
No. of available embryos	1.89 (1.63–2.20)**		1.23 (1.19–1.26)**		1.14 (1.08–1.20)**	
No. of high quality embryos	1.96 (1.68–2.28)**		1.26 (1.23–1.30)**	1.26 (1.05–1.50)*	1.15 (1.10–1.21)**	
Available embryo rate (%)	2.96 (1.69–5.20)**		10.34 (7.2–14.9)**		30.6 (8.7–107.0)**	
High quality embryo rate (%)	2.97 (1.80–4.90)**		12.8 (9.0–18.2)**		36.0 (10.5–123)**	
P level on the day of HCG	0.75 (0.56–1.00)*	0.60 (0.40–0.89)*	0.75 (0.67–0.84)**	0.76 (0.67–0.86)**	0.65 (0.48–0.88)**	0.66 (0.47–0.92)*

Note: BMI, body mass index; FSH, follicular stimulation hormone; Gn, gonadotropin; E_2_, estrogen; LH, luteinizing hormone; HCG, human chorionic gonadotropin; COR, curde odds ratio; AOR, adjusted odds ratio; CI, confidence interval.

^*^ P<0.05.

^**^ P<0.01.

### Intermediate ovarian responders

**Figure 2:**
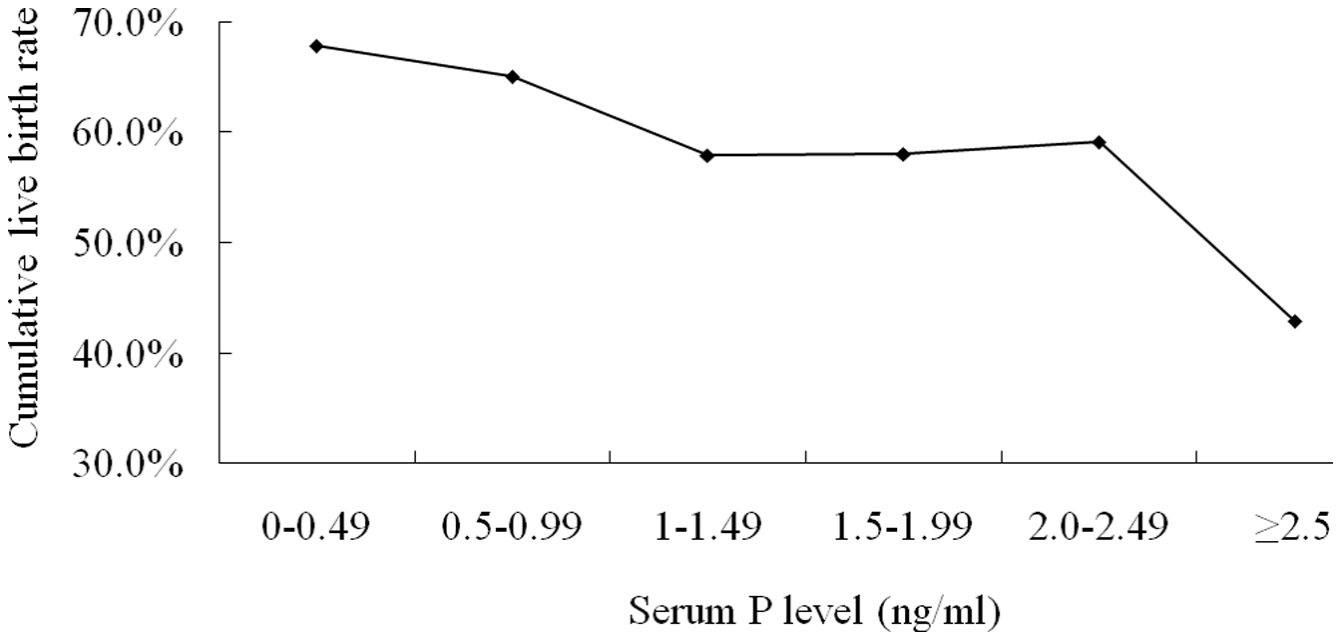
Relationship between serum P level on the day of HCG administration and cumulative live birth rate per oocyte retrieval cycle in intermediate ovarian responders (6≤ retrieved oocytes ≤19).

The relationship between cumulative live birth rate and serum P level in intermediate ovarian responders (n = 3,065) was shown in Figure 2. There was a reduction in cumulative live birth rate with progressively greater concentrations of serum P level. Compared with patients in non-elevation (P<2.24 ng/mL), those with P level elevation (P≥2.24 ng/mL) had significantly higher peak E_2_ levels, number of oocytes retrieved, and number of available embryos. Even though the fertilization rate, number of high quality embryos, available embryo rate, and high quality embryo rate were comparable between these two groups, the cumulative live birth rate was significantly lower in P level elevation patients compared with non-elevation patients (Table 5).

**Table 5:** Patients' characteristics and cycle outcomes in intermediate ovarian responders (6≤ retrieved oocytes ≤19) with and without P elevation (≥2.24 ng/ml) on day of HCG administration.

	Non-elevated P (n = 2912)	Elevated P (n = 153)	*P* value
Age (y)	30.4±4.5	31.6±4.3	0.001
BMI (Kg/m^2^)	22.2±3.0	22.0±2.9	NS
Duration of infertility (y)	4.6±3.2	5.1±3.4	0.041
Basal FSH (IU/L)	7.6±2.2	7.0±1.8	0.002
Gn duration (days)	11.2±2.0	11.4±2.2	NS
Gn dosage (IU)	2170±817	2705±942	0.000
Peak E_2_ (pg/ml)	5747±2862	7391±3403	0.000
Peak LH (IU/L)	2.0±1.2	1.7±3.0	NS
No. of retrieved oocytes	11.5±3.6	12.3±3.4	0.007
Fertilization rate (%)	68.1±18.8	68.8±18.6	NS
No. of available embryos	6.3±3.1	6.8±3.3	0.034
No. of high quality embryos	5.3±3.1	5.4±3.1	NS
Available embryo rate (%)	55.0±21.2	56.3±22.6	NS
High quality embryo rate (%)	46.1±22.6	44.3±22.9	NS
Cumulative live birth rate (%)	1837/2912 (63.1%)	72/152 (47.1%)	0.000

Note: values are the mean ± SD unless otherwise noted. NS, not significant.

BMI, body mass index; FSH, follicular stimulation hormone; Gn, gonadotropin; E_2_, estrogen; LH, luteinizing hormone.

Like the situation in poor ovarian responders, univariate logistic regression analysis in intermediate ovarian responders also showed that most parameters, including serum P level, pooled into the analysis were associated with cumulative live birth rate. However, in multivariate logistic regression analysis, serum P level was still significantly associated with cumulative live birth rate (Table 4).

### High ovarian responders

**Figure 3:**
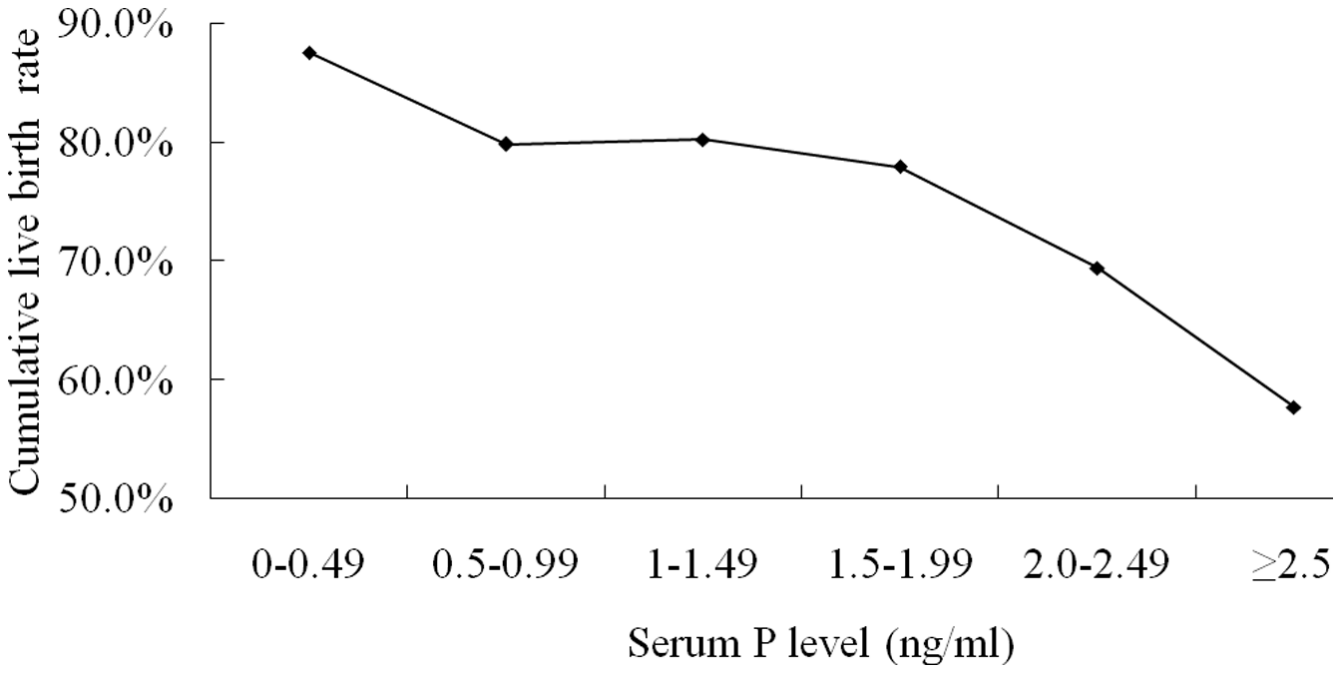
Relationship between serum P level on the day of HCG administration and cumulative live birth rate per oocyte retrieval cycle in high ovarian responders (retrieved oocytes ≥20).

For high ovarian responders (n = 482), the cumulative live birth rate also decreased with the increase of serum P level, especially when serum P level ≥2.50 ng/mL (Figure 3). Patients' basic information and clinical parameters are showed in table 5. The gonadotropin dose and peak E_2_ level were significantly higher in patients with serum P level elevation (P≥2.50 ng/mL). No differences were detected in the number of oocytes retrieved, fertilization rate, number of available embryos, number of high quality embryos, available embryo rate, and high quality embryo rate. However, the cumulative live birth rate was significantly lower in the group with serum P level elevation (Table 6).

**Table 6:** Patients' characteristics and cycle outcomes in high ovarian responders (retrieved oocytes ≥20) with and without P elevation (≥2.50 ng/ml) on day of HCG administration.

	Non-elevated P (n = 456)	Elevated P (n = 26)	*P* value
Age (y)	28.9±4.0	29.8±3.8	NS
BMI (Kg/m^2^)	22.3±3.1	21.2±2.0	NS
Duration of infertility (y)	4.2±2.9	5.0±3.3	NS
Basal FSH (IU/L)	6.6±1.6	6.4±1.5	NS
Gn duration (days)	11.0±1.6	10.9±2.6	NS
Gn dosage (IU)	1795±515	2343±760	0.000
Peak E_2_ (pg/ml)	9810±3530	12848±4360	0.000
Peak LH (IU/L)	1.5±0.7	1.2±0.8	NS
No. of retrieved oocytes	24.6±5.7	25.0±4.1	NS
Fertilization rate (%)	62.7±16.2	61.4±15.4	NS
No. of available embryos	12.0±4.9	12.8±5.1	NS
No. of high quality embryos	10.1±5.0	9.8±5.2	NS
Available embryo rate (%)	49.3±18.2	50.8±17.5	NS
High quality embryo rate (%)	41.6±19.5	39.2±19.3	NS
Cumulative live birth rate (%)	360/456 (78.9%)	15/26 (57.7%)	0.025

Note: values are the mean ± SD unless otherwise noted. NS, not significant.

BMI, body mass index; FSH, follicular stimulation hormone; Gn, gonadotropin; E_2_, estrogen; LH, luteinizing hormone.

By univariate logistic regression analysis, factors associated with cumulative live birth rate included duration of infertility, fertilization rate, number of available embryos, number of high quality embryos, available embryo rate, high quality embryo rate, and serum P level. The inverse association between serum P level and cumulative live birth rate remained significant after adjustment for confounding factors which was found to be associated with cumulative live birth rate by univariate analysis (Table 4).

## Discussion

Serum P elevation on the day of HCG administration, which was considered as premature luteinization in earlier studies, has been found to be more likely due to an accumulation from a large number of follicles [16]. Unlike most previous studies that simply assessed the relationship between serum P level and IVF/ICSI outcome in the overall patients, recent studies have realized that it is reasonable to divide patients into different groups according to ovarian response [10]. In addition, another study has shown that it is also important to explore the association between the duration of serum P elevation before HCG administration, but not just an absolute cutoff value of P level, and IVF/ICSI outcome [16]. However, irrespective of research methodology each study used, most scholars have reached a consensus on the adverse impact of serum P elevation on IVF/ICSI outcome.

From the patient's point of view, the most valuable parameter for them is the cumulative live birth rate per oocyte retrieval cycle, which is much similar as ‘taking baby home’ rate. For clinicians, cumulative live birth rate per oocyte retrieval cycle is also more meaningful as it gives us the opportunity to track the final clinical outcome by taking into account the outcomes of fresh as well as frozen embryos derived from the same oocyte retrieval cycle [17], [18]. The present large cohort study, which includes 4,651 patients undergoing their first IVF/ICSI cycle, demonstrates that cumulative live birth rate per oocyte retrieval cycle was significantly decreased in patients with serum P elevation, independent of the ovarian response.

It is known that endometrial receptivity and oocyte/embryo quality are two main factors associated with implantation [19]. The next question is how the elevated serum P level adversely affects IVF/ICSI outcome. Does serum P elevation only affect endometrial receptivity in fresh transfer cycles as most studies have been shown? Or the quality of oocytes resulting from the fresh cycles has also been compromised?

Among a large amount studies concerning the relationship between serum P elevation and IVF/ICSI outcome, some of them reported that the serum P elevation changes the implantation window rather than embryo quality, evidenced by oocyte donation cycles [20]. Most recently, a large retrospective study including more than 10,000 cycles also demonstrated no relationship between elevated P level and oocyte quality in all responders, as the fertilization rate, cleavage rate were comparable between P elevation and non-elevation groups. In addition, the study also showed that the ongoing pregnancy rate seemed to be much better in FET cycles than that in fresh cycles for women with elevated serum P level [10]. However, there was also a prospective study that directly showed high estrogen during IVF reducing implantation rate by affecting embryonic adhesion. High estrogen concentration, which also means high P levels as shown by our study, reduced both endometrial receptivity and embryo quality [21].

For all patients included into this study, the number of oocytes retrieved seemed to be higher in serum P elevation group compared with that in non-elevation group. However, the laboratory parameters in this study showed no better results for serum P elevation patients, especially in poor responders. What is more, the high quality embryo ratio was lower in serum P elevation group, although the difference did not reach statistical significant. All these results indicate that the oocyte quality may also be adversely affected by serum P elevation. In addition, the significantly reduced cumulative live birth rate in elevated serum P level patients with different response also indirectly supported this hypothesis.

There are also some limitations in the present study. Even though we evaluated the relationship between serum P level and cumulative live birth rate in different responders, which is an effective way to control for confounding factors, the retrospective nature of the study that may have led to bias in the interpretation of the data. Another limitation is that our data have no direct evidence to show the oocyte quality being adversely affected in serum P elevated patients. In this study, we used embryo morphology as our embryo scoring criteria, which may not really represent the ‘real quality’ of embryos [22], [23].

Taken together, our large retrospective, single-center study showed that serum P elevation on the day of HCG administration during IVF/ICSI-ET adversely affects the cumulative live birth rate per oocyte retrieval cycle in patients with different ovarian response. The effect of P elevation on embryo quality deserves more intensive study.
